# Case Report: Iliac perivascular myeloid sarcoma presenting with iliofemoral deep vein thrombosis and hydronephrosis

**DOI:** 10.3389/fonc.2026.1838693

**Published:** 2026-05-11

**Authors:** Zhao Shan, Yue Wang, Honggang Wang, Jiaxuan He, Hongyi Cai

**Affiliations:** 1 Department of Radiation Oncology, The First Clinical Medical College of Gansu University of Chinese Medicine (Gansu Provincial Hospital), Lanzhou, China; 2 Department of Radiation Oncology, Dongguan Taixin Hospital, Dongguan, China; 3 Department of Ultrasound Medicine, Wuwei People's Hospital, Wuwei, China

**Keywords:** acute myeloid leukemia, deep vein thrombosis, myeloid sarcoma, PET/CT, venetoclax

## Abstract

Myeloid sarcoma (MS) is a rare extramedullary tumor of myeloid blasts that may occur before, concurrently with, or after acute myeloid leukemia (AML) (1–4). We report a 53-year-old man with progressive left lower-limb pain and swelling, initially admitted to the vascular surgery service, with imaging revealing left external iliac vein and proximal common femoral vein occlusive thrombosis, a peri-iliac mass encasing the iliac vessels, and ipsilateral hydronephrosis. Biopsy of the left inguinal lesion showed MPO positivity, CD34 positivity, partial TdT positivity, and a Ki-67 index of about 80%, supporting myeloid sarcoma. Bone marrow studies confirmed AML with maturation, with 55% blasts, a myeloid immunophenotype, CEBPA (TAD1) positivity, and a complex karyotype. Baseline fluorine-18 fluorodeoxyglucose positron emission tomography/computed tomography (18F-FDG PET/CT) demonstrated irregular perivascular soft tissue extending from the left common to external iliac vessels, with mild FDG uptake. The patient received AML-directed therapy, including two IA cycles and high-dose cytarabine, achieving morphologic complete remission with a marked PET/CT response. During follow-up, measurable residual disease (MRD) fluctuated repeatedly despite continued morphologic remission, prompting venetoclax-based therapy, including cytarabine plus venetoclax, azacitidine plus venetoclax with homoharringtonine, and later azacitidine plus venetoclax combined with tislelizumab. At the last follow-up in December 2025, the patient remained alive. This case highlights that peri-iliac MS may mimic vascular and urologic disease and that serial MRD monitoring can be useful for guiding postremission treatment adaptation in AML with extramedullary disease.

## Introduction

Myeloid sarcoma (MS), also termed granulocytic sarcoma or chloroma, is an extramedullary accumulation of immature myeloid cells associated with myeloid neoplasms. MS may present concurrently with acute myeloid leukemia (AML), precede marrow disease as an isolated lesion, or represent relapse after prior therapy. Symptoms are mainly determined by the involved site, so MS often mimics more common surgical, vascular, infectious, or solid-tumor conditions, contributing to delayed diagnosis (1–4).

We report an uncommon case of peri-iliac MS presenting with iliofemoral deep vein thrombosis (DVT) and hydronephrosis in a 53-year-old man. The case is notable for a relatively comprehensive diagnostic workup, PET/CT response assessment, longitudinal measurable residual disease (MRD) fluctuations, and prolonged survival through December 2025.

## Case report

A 53-year-old man was admitted to the vascular surgery service on 9 December 2020 with progressive pain and swelling of the left lower limb for more than 1 year. Initial laboratory testing showed a normal complete blood count (WBC: 5.5 × 10^9^/L, hemoglobin: 149 g/L, platelet count: 279 × 10^9^/L), normal coagulation parameters, and normal liver and renal function, apart from a serum creatinine level of 105.62 µmol/L, with no relevant elevation of tumor markers. Abdominal ultrasonography showed left hydronephrosis and dilatation of the left mid-to-upper ureter. Duplex ultrasonography demonstrated occlusive thrombosis of the left external iliac vein and proximal common femoral vein, and a heterogeneous hypoechoic lesion encasing the iliac vessels in the left iliopsoas region (63 mm × 33 mm × 19 mm). Pelvic MRI confirmed an abnormal signal surrounding the left iliac vessels and enlarged left inguinal lymph nodes. CT pulmonary angiography showed no definite pulmonary embolism.

Biopsy of the left inguinal mass identified fragmented gray-white to gray-brown tissue, and immunohistochemical examination showed negative results for CKP, EMA, CD3, CD5, CD20, CD79a, HMB45, MelanA, CgA, Syn, P63, and CD56, with positive expression of myeloperoxidase (MPO) and CD34, partial TdT positivity, and a Ki-67 index of approximately 80%, consistent with myeloid sarcoma. Bone marrow morphology showed active hyperplasia with 55% blasts and positive peroxidase staining; peripheral blood blasts accounted for 9.0%. Bone marrow biopsy showed diffuse proliferation of primitive cells with MF-0 reticulin. Flow cytometry identified 51% blasts with a myeloid phenotype consistent with AML. Screening for 45 leukemia-related fusion transcripts was negative. WT1 was 15.50%, EVI1 was 0.04%, and CCAAT/enhancer-binding protein alpha (CEBPA) [transactivation domain 1 (TAD1) region] was positive. Cytogenetic analysis revealed a complex karyotype, including del(9)(q12q22), t(1;4)(p36;q12), t(1;7)(q21;q36), and loss of chromosomes 14, 17, and 20.

**Figure 1:**
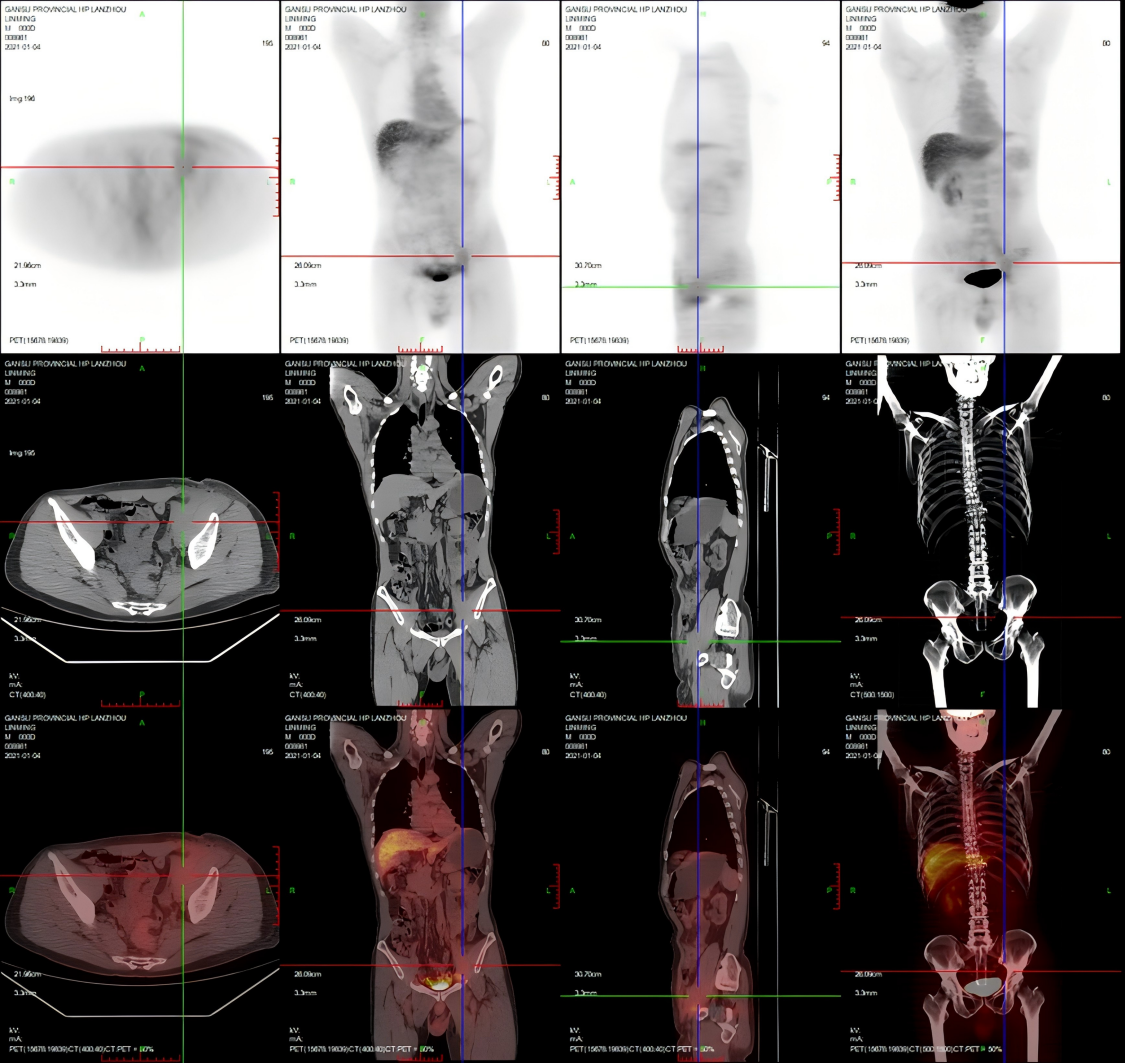
Baseline 18F-FDG PET/CT showing a peri-iliac perivascular soft-tissue lesion extending from the left common to external iliac vessels, with mild FDG uptake, compatible with myeloid sarcoma in the context of biopsy findings.

Baseline 18F-FDG PET/CT showed irregular strip-like soft tissue encasing the left common to external iliac vessels, with mildly heterogeneous FDG uptake (**Figure 1**), together with a small subcutaneous nodule in the left lower abdomen, findings considered compatible with peri-iliac MS. No other hypermetabolic lesion was identified. The patient was therefore diagnosed with AML with maturation (AML-M2), accompanied by peri-iliac myeloid sarcoma. The clinical timeline, MRD dynamics, and key management milestones are summarized in **Table 1**.

**Table 1:** Clinical timeline, MRD dynamics, and key management milestones.

Date	Key findings/interventions	Response/MRD
09 December 2020	Presentation with > 1-year left leg pain/swelling; left external iliac and proximal common femoral vein thrombosis; peri-iliac mass; ipsilateral hydronephrosis.	Diagnostic workup initiated
11 December 2020	Inguinal biopsy: MPO+, CD34+, partial TdT+, Ki-67 ~ 80%; marrow: 55% blasts, AML phenotype; CEBPA (TAD1)+; complex karyotype.	Diagnosis of AML-M2 with peri-iliac MS established
05 January 2021 to 18 February 2021	Two IA cycles.	Morphologic CR achieved; MRD remained positive
16 March 2021 to 19 March 2021	High-dose cytarabine and PET/CT reassessment.	MRD 0.074%; peri-iliac lesion nearly resolved; SUVmax 3.5
03 May 2021 to 07 June 2021	Further consolidation with high-dose cytarabine and cytarabine + idarubicin.	CR maintained; MRD later rose to 0.96%
16 August 2021 to 02 December 2021	Cytarabine + venetoclax, followed by repeat cycle.	MRD 0.11% after the first VEN-containing cycle, then negative; later rose again to 0.49%
December 2021 to September 2023	Venetoclax + azacitidine + homoharringtonine, then azacitidine + venetoclax + tislelizumab and venetoclax maintenance.	MRD decreased to 0.024%, later to 0.38%, and subsequently returned to negativity; the latest formal marrow assessment showed CR with peripheral blood MRD negative
December 2025	Last follow-up.	Alive; progression-free survival at least 59 months

The patient received first-cycle IA therapy on 05 January 2021 and a second IA cycle on 18 February 2021. High-dose cytarabine was then administered on 16 March 2021. Morphologic complete remission (CR) was achieved, but MRD remained detectable; the available record reported MRD at 0.074% after early postremission therapy. PET/CT reassessment on 19 March 2021 demonstrated near-complete regression of the previous peri-iliac lesion, with only mild residual FDG uptake (SUVmax: 3.5). High-dose cytarabine was administered on 03 May 2021, followed by cytarabine plus idarubicin on 07 June 2021, with MRD rising to 0.96%. Venetoclax-based therapy was introduced for recurrent MRD positivity. MRD remained positive at 0.11% after cytarabine plus venetoclax on 16 August 2021 and became negative after another cycle on 22 September 2021. MRD rose again to 0.49% on 2 December 2021, and the patient received venetoclax 400 mg orally plus azacitidine and homoharringtonine. Myelosuppression led to the temporary withholding of venetoclax. Neutropenic infection was treated with meropenem and tigecycline. MRD decreased to 0.024% and became negative 1 month later. Azacitidine plus venetoclax combined with tislelizumab was administered to the patient at multiple time points from March 2022 onward, including a documented 200-mg administration in June 2023. MRD became positive again at 0.38% in January 2023 but later returned to negativity. At the latest available formal assessment, bone marrow examination still showed CR, and peripheral blood MRD was negative. The patient remained alive at the last follow-up in December 2025; in the available record, progression-free survival was at least 59 months.

## Discussion

This case broadens the clinical spectrum of MS because peri-iliac disease first manifested as iliofemoral DVT and hydronephrosis rather than as an obvious hematologic malignancy. In patients with unexplained peri-vascular soft-tissue encasement, venous thrombosis, and adjacent urinary tract obstruction, MS should be considered in the differential diagnosis (1, 3, 4).

The diagnosis in this patient relied on concordant evidence from lesion pathology, marrow morphology, marrow biopsy, flow cytometry, cytogenetics, and PET/CT. Imaging alone was not diagnostic, but PET/CT helped define disease extent and later documented a marked response after AML-directed treatment. This supports the practical value of PET/CT in anatomically complex MS, particularly when lesions are difficult to assess by conventional imaging alone (5, 6).

The relevance of gene mutations and targeted therapies in MS has been increasingly recognized (7). Molecular and cytogenetic findings support the interpretation of this case as biologically systemic AML with extramedullary involvement, not a purely localized lesion. The patient had CEBPA (TAD1) positivity, complex karyotype, and recurrent MRD instability. The serial MRD course was clinically informative. MRD fell after induction and consolidation, later rose to 0.96%, decreased after venetoclax-based treatment, rose again, and subsequently became negative. These fluctuations indicate the morphologic remission alone did not fully capture residual disease burden and support the role of serial MRD monitoring in postremission decision-making.

From a therapeutic perspective, the patient achieved a favorable initial response to AML-type systemic chemotherapy, with morphologic CR and distinct PET/CT regression of the peri-iliac lesion. Venetoclax-based regimens were applied subsequently for recurrent MRD positivity despite persistent morphologic CR, including cytarabine plus venetoclax, azacitidine plus venetoclax combined with homoharringtonine, and azacitidine plus venetoclax with tislelizumab. Each regimen resulted in renewed MRD negativity at different time points. These observations correspond with limited existing literature supporting the potential activity of venetoclax-based regimens in AML with extramedullary disease, including MS (8, 9). This single case report cannot confirm the independent efficacy of each treatment component.

The PD-1 inhibitor-containing treatment phase requires cautious interpretation. Retrospective records confirm repeated administration of azacitidine plus venetoclax plus tislelizumab, with one documented dose of 200 mg. Biomarker evidence, including PD-L1 expression or T-cell infiltration, is unavailable. Allogeneic hematopoietic stem cell transplantation was discussed with the patient and family during treatment. Transplantation was not performed due to family refusal. The case remains valuable despite these limitations. It documents the rare presentation of peri-iliac MS with DVT and hydronephrosis, a multimodal diagnostic workflow, PET/CT response evaluation, and prolonged patient survival through December 2025.

## Patient perspective

The patient reported unilateral leg swelling and pain that significantly affected daily activities before diagnosis. Symptoms improved after systemic therapy. The patient consented to the publication of this case to raise awareness of rare extramedullary manifestations of AML.

## Data Availability

The original contributions presented in the study are included in the article/supplementary material. Further inquiries can be directed to the corresponding author.

## References

[1] Almond LM Charalampakis M Ford SJ Gourevitch D Desai A . Myeloid sarcoma: Presentation, diagnosis, and treatment. Clin Lymphoma Myeloma Leuk. (2017) 17:263–7. doi: 10.1016/j.clml.2017.02.027. PMID: 28342811

[2] Magdy M Abdel Karim N Eldessouki I Gaber O Rahouma M Ghareeb M . Myeloid sarcoma. Oncol Res Treat. (2019) 42:224–9. doi: 10.1159/000497210. PMID: 30840960

[3] Ramia de Cap M Chen W . Myeloid sarcoma: An overview. Semin Diagn Pathol. (2023) 40:129–39. doi: 10.1053/j.semdp.2023.04.009. PMID: 37149396

[4] Loscocco GG Vannucchi AM . Myeloid sarcoma: More and less than a distinct entity. Ann Hematol. (2023) 102:1973–84. doi: 10.1007/s00277-023-05288-1. PMID: 37286874 PMC10345021

[5] Irie S Inoue A Nakamura T Kobayashi Y Yamaguchi T Aoki R . Two cases of myeloid sarcoma of the mediastinum. Radiol Case Rep. (2025) 20:2063–9. doi: 10.1016/j.radcr.2025.01.035. PMID: 39944162 PMC11815513

[6] Liu W Yang X Chen LM Chen Y Xu TT . 18F-FDG PET/CT and 68Ga-DOTA-FAPI-04 PET/CT findings of myeloid sarcoma. Clin Nucl Med. (2023) 48:278–9. doi: 10.1097/rlu.0000000000004488. PMID: 36723889

[7] Fu L Zhang Z Chen Z Fu J Hong P Feng W . Gene mutations and targeted therapies of myeloid sarcoma. Curr Treat Options Oncol. (2023) 24:338–52. doi: 10.1007/s11864-023-01063-6. PMID: 36877373

[8] Padmanabhan DS Aguilar JJ Nanja Reddy S Shukla A Dhillon V Chohan S . Clinical and molecular characterization of myeloid sarcoma: A systematic review and meta-analysis. Cancers (Basel). (2025) 17:3975. doi: 10.3390/cancers17243975. PMID: 41463221 PMC12730967

[9] Tian FQ Zhang LS Li JH Tang MQ Jiang J Cheng XH . Venetoclax combined with azacitidine in the treatment of myeloid sarcoma: Three case reports and literature review. Zhonghua Xue Ye Xue Za Zhi. (2020) 41:694–6. doi: 10.3760/cma.j.issn.0253-2727.2020.08.016 PMC7525173 32942828

